# Spatiotemporal Distribution Shifts of *Zelkova schneideriana* Under Climate Change: A Biomod2-Driven Modeling Framework

**DOI:** 10.3390/biology14091221

**Published:** 2025-09-08

**Authors:** Mimi Li, Lingdan Wang, Hailong Liu, Yueqi Sun, Naiwei Li, Maolin Geng

**Affiliations:** 1 Institute of Botany, Jiangsu Province and Chinese Academy of Sciences, Nanjing 210014, China; mli@jib.ac.cn (M.L.);; 2 Jiangsu Key Laboratory for Conservation and Utilization of Plant Resources, Nanjing 210014, China; 3 School of Civil Engineering, Central South University of Forestry and Technology, Changsha 410004, China; 4 Guangxi Key Laboratory of Superior Timber Trees Resource Cultivation, Guangxi Forestry Research Institute, Nanning 530002, China

**Keywords:** biomod2, climate change, ensemble model, glacial refugia, *Zelkova schneideriana*

## Abstract

*Zelkova schneideriana* (Ulmaceae), an endemic relict species of the Tertiary in China, has experienced a sharp decline in population due to habitat fragmentation, poor natural regeneration, and anthropogenic disturbances. It is currently listed as a category II national key protected wild plant and categorized as Vulnerable by the International Union for Conservation of Nature (IUCN). To explore its response mechanisms to climate change, this study integrates 11 species distribution models (SDMs) to comprehensively predict its suitable habitat distribution patterns. Key environmental variables were identified as Bio06 (minimum temperature of the coldest month, 21.57%), Bio02 (mean diurnal range, 19.81%), Bio17 (precipitation of the driest quarter, 13.52%), Bio15 (precipitation seasonality, 8.32%), Bio07 (temperature annual range, 8.15%), Bio12 (annual precipitation, 6.58%), and elevation (6.57%), collectively contributing approximately 85%. Spatiotemporal analysis revealed that during historical glacial periods, suitable habitats were significantly restricted, and highly suitable zones were absent under extreme climatic conditions, suggesting the presence of potential glacial refugia. Under current climatic conditions, highly suitable habitats have expanded notably. However, under the high-emission scenario (SSP585) in the future, the suitable range is projected to shrink considerably, with a drastic reduction in highly suitable areas. Moreover, the suitability centroid is expected to shift markedly toward higher elevations in the northeast, indicating a potential adaptation strategy of *Z. schneideriana* toward mountainous regions in Hunan, Hubei, and Chongqing. These findings provide quantitative guidance for the formulation of targeted conservation strategies for *Z. schneideriana* and offer methodological insights for predicting suitable habitats and managing related relict plant species under the threat of climate change.

## Simple Summary

*Zelkova schneideriana*, a vulnerable tree species native to China, is facing a decline in population. This study employed the ensemble model biomod2 to predict the species’ suitable habitats and how these habitats might change during past and future climate scenarios. The results highlight the significance of key environmental factors, such as temperature, precipitation, and elevation, in determining suitable habitats. During past periods, its habitat was limited, with no highly suitable areas under extreme conditions. At present, the habitat has significantly expanded. However, under future high-emission scenarios, its suitable habitat is expected to contract and shift toward regions at higher elevations with cooler temperatures. These insights offer critical guidance for the conservation and adaptive management of *Z. schneideriana* and other species in the face of climate change.

## 1. Introduction

*Zelkova schneideriana* Hand.-Mazz., a Tertiary relict species in the family Ulmaceae, is an endemic and precious tree species in China. It is primarily distributed in subtropical regions and adjacent mountainous transition zones, possessing both ecological and economic value [1]. As a fast-growing species with significant ornamental appeal, *Z. schneideriana* serves as a high-quality resource for landscaping and urban greening. Its timber, characterized by beautiful grain and strong decay resistance, is favored for high-level furniture and decorative uses. Due to a sharp decline in natural populations, *Z. schneideriana* has been listed as a category II national key protected wild plant and was assessed as Vulnerable (VU) by the International Union for Conservation of Nature (IUCN) in 2018 [2].

*Z. schneideriana* faces dual survival pressures: resource degradation caused by overexploitation and weak natural regeneration. Meanwhile, climate change is profoundly altering its habitat through shifts in temperature and precipitation regimes. Habitat contraction, degradation, and loss pose a severe threat to *Z. schneideriana*, which exhibits patchy distribution and limited dispersal ability, potentially accelerating its extinction risk. To quantify these impacts, species distribution models (SDMs) integrate historical and current occurrence data with climate variables to simulate ecological niche requirements and assess environmental adaptability. Multi-temporal distribution modeling facilitates the understanding of historical shifts, current distribution patterns, and the mechanisms underlying species responses to climate change.

Previous studies have used ecological niche models to explore the response patterns of *Z. schneideriana* to climate change. For instance, Sun et al. [3] applied the MaxEnt model and found that *Z. schneideriana* is undergoing a northward distribution shift due to anthropogenic activity and climate impacts, with future habitat suitability mainly decreasing, and limited expansion potential. Bio17 (precipitation of the driest quarter) was identified as a key limiting factor. Zhou et al. [4], by comparing *Z. schneideriana* with its associated species (*Phyllostachys edulis* and *Celtis sinensis*), further confirmed that temperature-related factors play a dominant role in determining its distribution, predicting a decrease in suitable area and a northeastward shift in the suitability centroid. Additionally, He et al. [5] found that solar radiation in May is a key environmental driver for the survival of ancient *Z. schneideriana* individuals in Hunan Province. Notably, previous studies rely on a single MaxEnt model, and the identified key environmental variables vary, limiting the precision of conservation planning. Accurate identification of core environmental drivers is thus critical for effective monitoring and management strategies.

Ensemble modeling frameworks, which can reduce uncertainty and improve predictive robustness, offer significant advantages in SDM studies [6]. By integrating the outputs of multiple algorithms, ensemble models produce more stable and reliable results, minimizing performance fluctuations caused by variations in input data. Biomod2, a comprehensive SDM platform, enhances the reliability of species distribution predictions by integrating multiple models and evaluation metrics. It also allows users to define filtering criteria among successfully run models, enabling the selection of individual models that meet specific performance thresholds for constructing ensemble predictions [7]. This approach has been widely used for predicting species’ suitable habitats, potential range shifts, and responses to climate change [8,9,10,11]. In particular, such models are valuable in endangered species conservation, providing a scientific basis for reserve delineation and ex-situ conservation strategies [12,13].

Given this context, the present study employs the biomod2 ensemble modeling framework to systematically analyze the spatiotemporal dynamics of *Z. schneideriana*, aiming to address the following questions: (1) What are the key environmental factors driving its geographical distribution? (2) How do habitat suitability patterns shift across different climate change scenarios? (3) Based on model projections, what adaptive conservation and restoration strategies can be proposed, especially for vulnerable and potentially expandable areas? By elucidating the mechanisms through which climate change influences the distribution of *Z. schneideriana*, this study provides critical scientific support for the species’ conservation, sustainable management, and biodiversity protection under global environmental change.

## 2. Materials and Methods

### 2.1. Data Sources and Processing

A total of 483 distribution records of *Z. schneideriana* were obtained by compiling data from multiple digital platforms, including the Global Biodiversity Information Facility (GBIF, http://www.gbif.org, accessed on 19 February 2025), the Chinese Virtual Herbarium (CVH, https://www.cvh.ac.cn, accessed on 19 February 2025), the National Specimen Information Infrastructure (NSII, http://www.nsii.org.cn, accessed on 19 February 2025), the Teaching Specimen Resource Sharing Platform (http://mnh.scu.edu.cn/index, accessed on 19 February 2025), and the Plant Photo Bank of China (PPBC, https://ppbc.iplant.cn, accessed on 19 February 2025), as well as from literature sources and field survey data. To reduce spatial autocorrelation while preserving data integrity, records of artificial cultivation and duplicates were removed. The R v4.5 package dismo v1.3 [14] was used to reduce sampling bias by filtering out duplicated and closely located points. Only one occurrence was retained within each 5 km × 5 km grid cell, resulting in 321 valid distribution records for subsequent modeling.

### 2.2. Selection and Processing of Environmental Variables

Modeling was conducted across seven temporal periods: the Last Interglacial (LIG, ~130,000 years ago), Last Glacial Maximum (LGM, ~21,000 years ago), Middle Holocene (MH, ~6000 years ago), the present period (1970–2000), and three future projection periods: 2050s (2041–2060), 2070s (2061–2080), and 2090s (2081–2100).

At broad spatial scales, climate is generally recognized as the dominant factor influencing species distributions [15]. Nineteen bioclimatic variables (Bio01–Bio19) and elevation were obtained from the WorldClim v2.1 database (http://www.worldclim.org/, accessed on 15 May 2024). For historical climate scenarios, the CCSM4 model, which is widely recognized for accurately simulating plant distributions, was adopted. Future climate projections were derived from the BCC-CSM2-MR model developed by the Beijing Climate Center, suitable for simulating species habitats in China, and part of the Coupled Model Intercomparison Project Phase 6 (CMIP6) [16]. Future climate scenarios were based on the Shared Socioeconomic Pathways (SSPs) from the IPCC sixth assessment report [17], including SSP126 (low emissions and sustainable development), SSP245 (medium emissions), and SSP585 (high emissions).

Slope and aspect data were derived from the Digital Elevation Model (DEM), and Chinese maps were sourced from the National Fundamental Geographic Information System (NFGIS, https://www.tianditu.gov.cn, accessed on 15 May 2024). All environmental layers were resampled to a spatial resolution of 2.5 arc-minutes (~5 km). For the LIG period, which had an original resolution of 30 arc-minutes, the data were resampled to 2.5 arc-minutes using the R v4.5 package terra v1.8 [18].

To minimize the effect of multicollinearity among the 22 environmental variables on model accuracy, Pearson correlation coefficients were calculated using the R v4.5 package corrplot v0.95 [19].

### 2.3. Model Construction

Species distribution modeling was conducted using the R v4.5 package biomod2 v4.2 [20]. Eleven algorithms were employed: Artificial Neural Networks (ANNs), Classification Tree Analysis (CTA), the Generalized Boosting Model (GBM), the Generalized Linear Model (GLM), Multivariate Adaptive Regression Splines (MARS), Maximum Entropy (MAXENT), Maximum Network (MAXNET), Random Forest (RF), the Surface Range Envelope (SRE), and eXtreme Gradient Boosting (XGBOOST). Model accuracy was assessed using the Area Under the Receiver Operating Characteristic (ROC) Curve (AUC) [21] and the True Skill Statistic (TSS) [22]. Models were trained on 75% of the data using stratified random sampling, while the remaining 25% was used for validation.

AUC measures performance across all thresholds, with values closer to 1 indicating better model performance: 0.9–1.0 indicates excellent, 0.8–0.9 indicates good, 0.6–0.7 indicates poor, and below 0.6 suggests model failure [23]. TSS accounts for omission and commission errors, as well as the success of random prediction, with the following classifications: excellent (TSS > 0.8), good (0.6–0.8), moderate (0.4–0.6), poor (0.2–0.4), and failed (TSS < 0.2) [22].

A total of 10,000 pseudo-absence points were randomly generated. Models with TSS values below 0.7 were excluded. Ensemble modeling was then performed based on TSS values using the BIOMOD_EnsembleModelling function, which provides four integration options via the em.algo parameter: EMmean (mean probability), EMmedian (median probability), EMca (committee averaging), and EMwmean (weighted mean).

### 2.4. Distribution Area Classification and Change Analysis

Habitat suitability was classified into four categories using the Jenks natural breaks method: unsuitable (0–0.2), low suitability (0.2–0.5), moderate suitability (0.5–0.7), and high suitability (0.7–1.0) [24]. The total area of each suitability category was calculated. A threshold of 0.2 was applied to convert suitability maps into binary suitable–unsuitable layers. SDMtoolbox v2.6 [25] was utilized to spatially overlay current patterns with historical and future scenarios. This approach quantified range contractions or expansions under shifting climates. Additionally, the suitable areas in each period were converted from raster to vector polygons, and their mean centers were calculated to obtain the centroids. The resulting centroid trajectories illustrate the spatiotemporal dynamics of suitable habitat distribution for *Z. schneideriana*.

## 3. Results

### 3.1. Model Accuracy and Performance Evaluation

**Figure 1:**
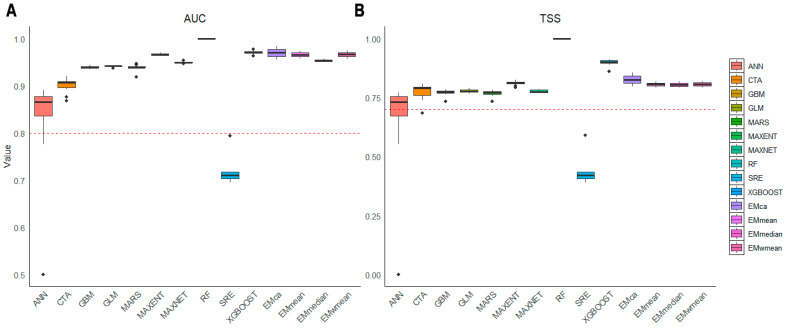
AUC and TSS evaluation of the model accuracy. (**A**), AUC; (**B**), TSS. ANN, artificial neural networks; CTA, classification tree analysis; GBM, generalized boosting model; GLM, generalized linear model; MARS, multivariate adaptive regression splines; MAXENT, maximum entropy; MAXNET, maximum net; RF, random forest; SRE, surface range envelope; XGBOOST, extreme gradient boosting. EMmean (mean), EMmedian (median), EMca (committee averaging), and EMwmean (weighted mean).

Among the 11 evaluated model types, AUC values (0.72–1) and TSS values (0.44–1) indicated substantial differences in modeling capability. Artificial Neural Networks (ANNs) and the Surface Range Envelope (SRE) performed relatively poorly, whereas eXtreme Gradient Boosting (XGBOOST) and Random Forest (RF) achieved the best performance. Based on the TSS > 0.7 criterion, ensemble models constructed using EMmean, EMmedian, EMca, and EMwmean demonstrated excellent performance, with AUC ranging from 0.954 to 0.966 and TSS from 0.804 to 0.811. The results confirm the robustness of ensemble methods for species distribution modeling. In particular, EMca yielded the highest accuracy; therefore, this method was selected for all subsequent ensemble modeling analyses (Figure 1). The ensemble model provides a reliable data foundation for assessing the climatic suitability of *Z. schneideriana*.

### 3.2. Dominant Environmental Factors

**Figure 2:**
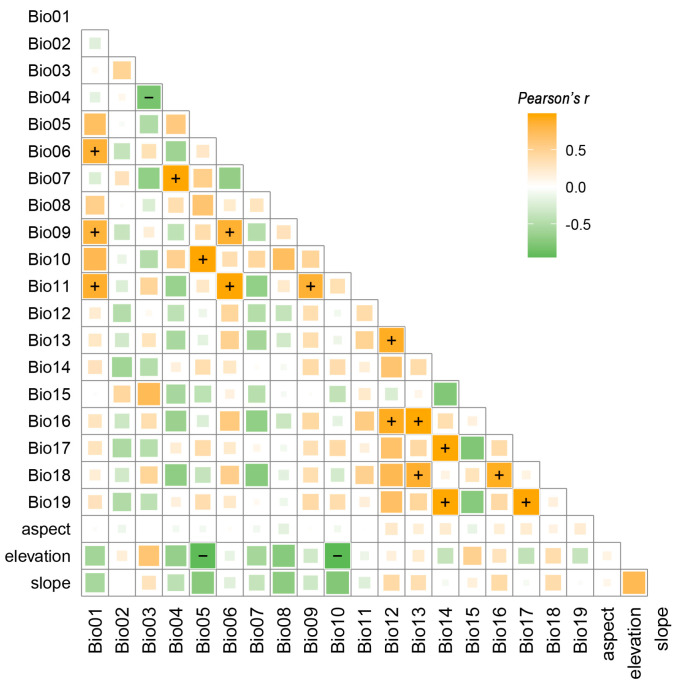
Correlation plot of environmental variables. + indicates a correlation greater than 0.8, and − indicates a correlation less than −0.8.

Environmental factors with correlation coefficients greater than |0.8| were removed (Figure 2), resulting in the retention of 12 high-contribution variables for predicting the potentially suitable habitat of *Z. schneideriana* (Table 1).

Analysis of environmental variable contributions revealed significant differences in the key factors determining the suitable distribution of *Z. schneideriana* (Table 1). The dominant variables were Bio06 (minimum temperature of coldest month, 21.57%), Bio02 (mean diurnal range, 19.81%), Bio17 (precipitation of driest quarter, 13.52%), Bio15 (precipitation seasonality, 8.32%), Bio07 (temperature annual range, 8.15%), Bio12 (annual precipitation, 6.58%), and elevation (6.57%), collectively accounting for approximately 85% of total contribution [26]. Overall, temperature-related variables (total weight 49.53%) exerted a greater influence on distribution patterns than precipitation-related variables (total weight 28.42%). Additionally, Bio18 (precipitation of warmest quarter) and Bio03 (isothermality) also contributed (>5%), whereas slope, Bio08 (mean temperature of wettest quarter), and aspect had relatively low contributions, exerting minimal influence on suitability.

### 3.3. Spatiotemporal Dynamics of Potentially Suitable Areas

**Figure 3:**
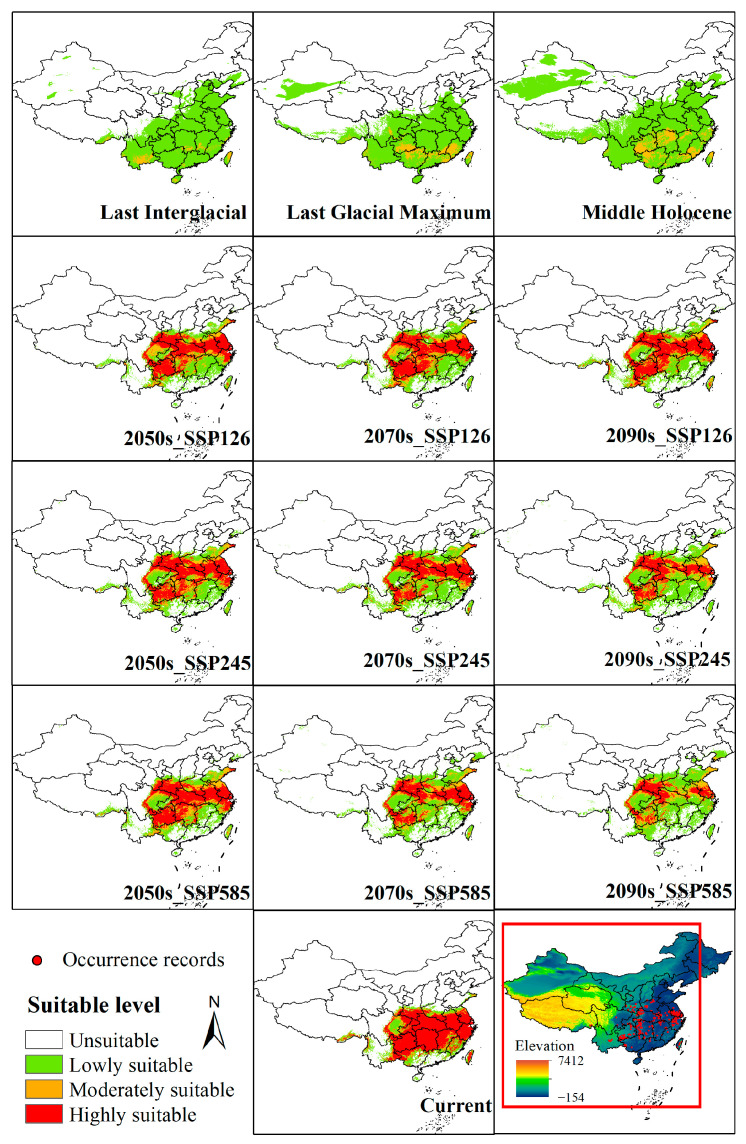
Distribution of potentially suitable habitats of *Z. schneideriana* at different periods. The red box indicates the area shown in other maps.

The suitable distribution range of *Z. schneideriana* shows significant changes across different periods and climate scenarios (Table 2, Figure 3). In historical periods, during the Last Interglacial (LIG) and Last Glacial Maximum (LGM), harsh climatic conditions resulted in the dominance of low-suitability areas (280.84 × 10^4^ km^2^ and 274.54 × 10^4^ km^2^, respectively). In the LIG, moderately suitable areas were only patchily distributed in southern Tibet, southern Yunnan, southern Hunan, southern Jiangxi, and northern Taiwan. The Hengduan Mountains in southwestern China were primarily characterized by warm and humid conditions during the LIG, but complex topography and microclimatic heterogeneity led to fragmented survival of *Z. schneideriana*, limiting its continuous distribution [27]. By the LGM (average annual temperature drop of 4–7 °C), the core areas of moderate suitability had shifted to Fujian, Guangxi, Guangdong, and Guizhou provinces, while highly suitable areas were entirely absent throughout the glacial stages, confirming the strong constraints of glacial climates on the survival of *Z. schneideriana*. In the Middle Holocene (MH), the total suitable area increased markedly (418.48 × 10^4^ km^2^), with suitability mainly concentrated in low to moderate levels. Moderate suitability areas expanded, but highly suitable areas were still absent, indicating that although climatic constraints had eased, they had not yet reached the optimal threshold.

**Figure 4:**
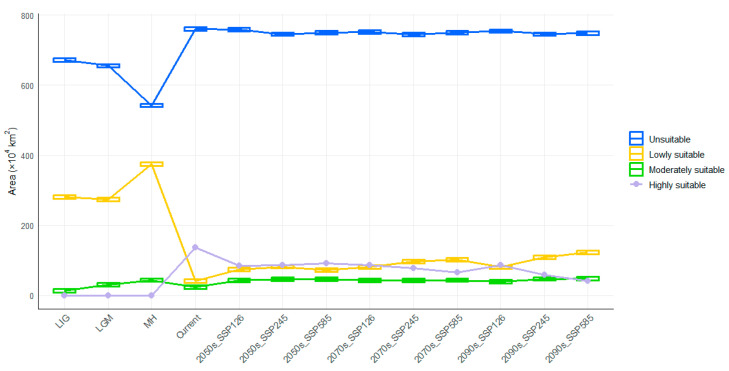
Potential suitable habitat area of *Z*. *schneideriana*.

At present, highly suitable areas cover 136.82 × 10^4^ km^2^, indicating the highest suitability of *Z. schneideriana* under modern climatic conditions. Hunan, Hubei, Guizhou, Jiangxi, Anhui, Jiangsu, Chongqing, Taiwan, and surrounding regions represent the main highly suitable areas, while Tibet, Guangdong, and Hainan show scattered distributions. Fujian, Guangxi, and Sichuan are the primary moderately suitable regions (Figure 4).

**Figure 5:**
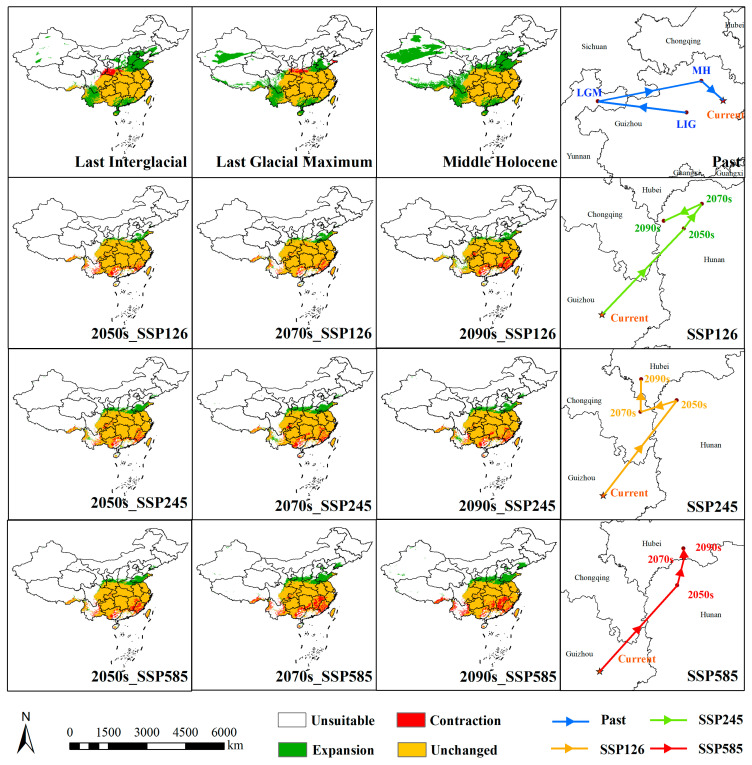
Changes in the potentially suitable habitat area and centroid of *Z. schneideriana* at different scenarios.

Future simulations under different SSP climate scenarios indicate pronounced spatial dynamics. From the present to future periods, stable (unchanged) areas remain extensive, ranging from 182.56 × 10^4^ km^2^ to 202.69 × 10^4^ km^2^. Expansion areas increase progressively over time, reaching 42.75 × 10^4^ km^2^ under the SSP585 scenario in the 2090s, suggesting that climate change may drive *Z. schneideriana* into new regions. However, there are also significant contractions, with highly suitable areas in Hunan, Jiangxi, and Hubei shrinking sharply, particularly under the SSP245 and SSP585 scenarios. The maximum contraction reaches 35.31 × 10^4^ km^2^, highlighting the directional influence of climate scenarios on the species distribution pattern. By the 2090s, under SSP585, the highly suitable area is reduced to be 41.37 × 10^4^ km^2^, while low-suitability areas increase to 122.74 × 10^4^ km^2^, indicating that high-emission pathways will lead to habitat quality degradation (Table 2, Figure 5).

The spatiotemporal migration characteristics of the distribution centroid of *Z. schneideriana* (Figure 5) show that during the LIG, the centroid was located at the border between Guiyang and Qiannan in Guizhou Province (107.21° E, 27.24° N), at an elevation of 660 m. In the LGM, the centroid shifted 286 km west to Zhaotong, Yunnan Province (104.34° E, 27.61° N), with elevation increasing to 1768 m. The upward shift in elevation corresponds to the species’ adaptation to glacial climatic conditions, wherein high-altitude areas provided relatively cooler and more humid climates that mitigated environmental stress. This response is commonly observed among relict temperate tree species that persisted through glacial cycles. In the MH, there was a significant eastward shift of 336 km to Zunyi (107.68° E, 28.27° N, 1152 m), followed by a further migration to the modern centroid in Tongren (108.39° E, 27.64° N, 860 m, 98 km away). During post-glacial warming conditions, *Z. schneideriana* demonstrated a tendency to shift toward lower elevations, where milder temperatures and improved moisture availability contributed to expanded distribution. This pattern reflects an ecological niche relaxation in response to more favorable climatic conditions. Under future scenarios, the centroid continues to migrate northeastward. In the SSP126 scenario, the centroid moves to northwestern Hunan Province in the 2050s, reaching Xiangxi Prefecture (109.69° E, 29.01° N, 608 m), then shifts northward to Zhangjiajie in the 2070s (109.97° E, 29.40° N, 362 m), with elevation decreasing by 498 m compared to the present, and returns to Xiangxi in the 2090s (109.37° E, 29.13° N, 740 m), with a cumulative migration distance of 315 km. In the SSP245 scenario, more significant inter-provincial migration occurs, with the centroid in the 2050s moving 220 km northeast to Xiangxi (109.66° E, 29.28° N, 707 m), shifting westward to Chongqing in the 2070s (109.03° E, 29.08° N, 514 m), and further north to Enshi, Hubei, in the 2090s (109.04° E, 29.64° N, 788 m). In the SSP585 scenario, the migration range is largest, starting in low-elevation Xiangxi in the 2050s (109.87° E, 29.28° N, 465 m) and moving into the higher elevations of Enshi in the 2070s and 2090s (2070s: 110.00° E, 29.80° N, 997 m; 2090s: 109.99° E, 29.99° N, 988 m). Overall, the results reveal a migration trajectory of *Z. schneideriana* from the mid to high-elevation regions of the southwestern Yunnan–Guizhou area toward the mid to low-elevation regions of Hunan, Hubei, and Chongqing, with migration distance and elevation range increasing with higher greenhouse gas emissions, suggesting an adaptive retreat toward higher elevations under high-emission scenarios.

## 4. Discussion

### 4.1. Model Evaluation

Species distribution models (SDMs) vary widely in both conceptual framework and algorithmic approach, resulting in complementary strengths and limitations among different modeling methods. Previous studies have shown that an overreliance on a single modeling method can significantly constrain predictive performance, as even when applied to the same dataset, different models often yield markedly divergent results. In the absence of a complete understanding of the true relationships between species and environmental changes, the use of a single model increases the risk of predictive bias [28].

To improve predictive reliability, this study employed an ensemble modeling framework to simulate the ecological niche of *Z. schneideriana* and analyze the spatiotemporal dynamics of its potentially suitable habitats. The ensemble model, constructed from bioclimatic and topographic variables, exhibited strong predictive performance, with AUC values ranging from 0.954 to 0.966 and TSS values from 0.804 to 0.811. These results indicate high robustness and the ability to deliver more comprehensive and accurate habitat predictions, thereby supporting a more precise analysis of the principal environmental drivers shaping the potential distribution of *Z. schneideriana*.

### 4.2. Environmental Factors

Multiple factors influence species distribution, with climate, especially precipitation and temperature, being the key drivers at broad spatial scales [29]. Climatic conditions influence all stages of a species’ life cycle [7], exerting significant effects on growth, survival, and reproduction, thereby shaping spatial distribution patterns and potential habitat suitability [30].

Our findings indicate that temperature- and precipitation-related variables dominate the distribution suitability of *Z. schneideriana*. Specifically, the minimum temperature of the coldest month (Bio06), the mean diurnal range (Bio02), and the precipitation of the driest quarter (Bio17) showed the highest contributions, suggesting that the species is sensitive to extreme cold and drought and has limited tolerance to such conditions. The sensitivity restricts its expansion into higher latitudes or arid areas. In addition, precipitation seasonality (Bio15) and temperature annual range (Bio07), which reflect interannual climate variability, also significantly influenced the distribution. Among all environmental variables, Bio06, representing the minimum temperature of the coldest month, was identified as the most important variable, aligning with the results of Zhou et al. [4], which also identified extreme winter cold as the primary limiting factor for the species range. These patterns suggest that *Z. schneideriana* has a relatively low threshold of physiological tolerance to cold and climatic fluctuations. The dominant influence of temperature of *Z. schneideriana* exceeds that of other variables, which may be due to its evolutionary adaptation as a Tertiary relict species in refugia [31].

Although precipitation variables contributed to the model, the species’ primary occurrence in humid regions suggests that precipitation is not the main limiting factor [32]. This differs from the findings of Sun et al. [3], who identified precipitation as the most important environmental driver. The discrepancy may be due to differences in sampling extent: our study covers a broader ecological range from the warm temperate to subtropical zones of the Yangtze River Basin, while Sun et al. [3] included ecologically atypical sites. This highlights the importance of geographical sampling choices in interpreting species–environment relationships.

Among topographic variables, elevation was a key determinant of *Z. schneideriana* distribution, reflecting its ecological preference for complex mountainous terrains. Although slope, aspect, and mean temperature of the wettest quarter (Bio08) contributed relatively little overall, they may still exert important moderating effects in specific topographies or microhabitats [33], with ecological significance at finer scales.

### 4.3. Spatiotemporal Dynamics of Distribution

Crucially, the cyclical oscillations between glacial and interglacial periods since the Quaternary have profoundly shaped modern species distributions [34]. In East Asia, the absence of extensive ice sheet coverage allowed the persistence of numerous ancient lineages [35,36]. From the Last Interglacial (LIG) to the Last Glacial Maximum (LGM), the distribution centroid of *Z. schneideriana* shifted northwestward, with a significant rise in elevation, indicating that severe glacial climates forced the species to contract into glacial refugia at lower latitudes and higher elevations. The resulting habitat compression suggests that *Z. schneideriana* likely survived in geographically restricted areas with relatively mild climates. During the LGM, the main refugia of *Z. schneideriana* were located in the Hengduan Mountains, the Yunnan–Kweichow Plateau, the Nanling Mountains, Wuyi Mountain, and the Central Mountain Range of Taiwan. These areas overlap with the glacial refugia of other East Asian Tertiary relict plants, such as *Sargentodoxa cuneata* [37], *Castanopsis carlesii* [38], and *Cyclobalanopsis glauca* [39], and other East Asian Tertiary relics [40]. These refugia played a crucial role in maintaining population continuity during extreme climatic events and may have been significant sources of modern genetic and species diversity [41,42,43,44,45,46]. Long-term geographic isolation and local adaptive evolution may have led to the development of unique genetic lineages in these regions [47,48,49,50], which could form the foundation for the genetic structure of *Z. schneideriana*. Thus, identifying and protecting these key areas is critical for understanding the evolutionary history of endangered plants [51]. It also provides clear spatial references for prioritizing conservation efforts under future climate change scenarios.

By contrast, the relative stability of the modern climate has allowed *Z. schneideriana* to occupy a broader and more continuous ecological space, with highly suitable areas now covering 136.82 × 10^4^ km^2^, reflecting a more complete expression of its ecological niche. The expansion pattern suggests that modern conditions have facilitated ecological release and enabled range expansion. The continuous and expanded distribution reduces isolation-by-distance effects and promotes gene flow through increased pollen and seed dispersal across broader landscapes, potentially enhancing heterozygosity and allelic richness, thereby boosting the population’s adaptive potential [52]. Molecular studies of Tertiary relict species suggest that such expansions can enhance resilience to environmental stress. However, fragmented remnants in peripheral areas may still harbor unique alleles from glacial refugia, necessitating integrated genetic monitoring to assess overall diversity gains [53,54].

However, with future warming, the species’ suitability pattern will undergo significant shifts, with suitable habitats in China migrating northeastward and expansion areas increasing over time, reaching 42.75 × 10^4^ km^2^ by the 2090s under the SSP585 high-emission scenario. This suggests that warming may facilitate the colonization of new areas. At the same time, contraction is pronounced, particularly in highly suitable areas of Hunan, Jiangxi, and Hubei, with the SSP585 scenario reducing these habitats to 41.37 × 10^4^ km^2^ by the 2090s, reflecting severe habitat degradation under high emissions, a finding that aligns with previous studies [3,4]. In all three future scenarios, the distribution centroid consistently shifts northeast, tracing migration from the southwestern Yunnan–Kweichow Plateau toward mid- to low-elevation areas in Hunan, Hubei, and Chongqing, with both the direction and magnitude of migration intensifying under higher greenhouse gas emissions. This migration pattern aligns with other subtropical plant species [37,55] and indicates an adaptive strategy of retreat to higher-elevation refugia under high-emission scenarios [56,57,58], highlighting both the species’ sensitivity to habitat changes and the need for future conservation strategies to focus on dynamic habitat management along elevational gradients and the designation of flexible protection zones.

### 4.4. Species Conservation and Restoration Strategies

The simulation of the distribution of *Z. schneideriana* provides valuable insights for conservation and restoration strategies. Future efforts should focus on identifying and protecting historical refugia and currently stable, suitable areas as core regions to maintain genetic diversity and evolutionary potential. It is crucial to monitor genetic exchanges and adaptive trait evolution in expanding populations to establish a dynamic baseline for long-term conservation [59]. Priority should be given to strengthening ecological connectivity across administrative boundaries [60], particularly in overlapping areas between current and future suitable habitats, to reduce the risk of population isolation under high radiative forcing scenarios.

Conservation-oriented restoration should be implemented in highly suitable areas [61], with reintroductions not only within the species’ current or recent range but also into areas projected to become suitable in the future based on modeling results. Establishing mixed-species forests composed of locally adapted species can further improve ecosystem resilience [62]. Given the limited research progress on *Z. schneideriana*, there is an urgent need to advance studies in its conservation biology, especially on seed germination and seedling growth responses under temperature gradient stress, to better understand its adaptation to thermal changes and the mechanisms underlying its stress resistance [63]. The modeling framework employed in this study can also serve as a reference for evaluating conservation strategies for related species across East Asia.

### 4.5. Limitations

Although this study employed an integrated modeling framework and utilized multiple algorithms through the biomod2 platform to generate robust and accurate predictions for the distribution of *Z. schneideriana*, several limitations remain. First, the selection of environmental variables primarily focused on climate and topographic factors, neglecting non-climatic influences such as land-use changes, human activities, and biological factors, which may have led to an underestimation of their impact on habitat suitability. Additionally, the redundancy removal method relied solely on Pearson’s correlation coefficient and did not account for non-linear collinearity, potentially resulting in the retention of redundant variables or the omission of key ones. In terms of climate model selection, relying on a single model may introduce systematic errors, affecting the accuracy of habitat change comparisons. Therefore, future research should focus on improving the diversity of environmental variables, refining climate model selection, and further exploring biological mechanisms and genetic diversity to provide more robust and comprehensive species distribution predictions and conservation strategies.

## 5. Conclusions

This study systematically integrated 11 species distribution models and employed a high-accuracy ensemble model to simulate the suitable habitat distribution of *Z. schneideriana*. Analysis of key environmental variables revealed that temperature-related factors, particularly the minimum temperature of the coldest month and mean diurnal range, exerted a stronger influence on the species distribution than precipitation variables, with elevation also playing an important role. Spatiotemporal patterns indicated that during past glacial periods (LIG and LGM), harsh climatic conditions led to the disappearance of highly suitable habitats, with the Hengduan Mountains, Yunnan–Kweichow Plateau, Nanling Mountains, Wuyi Mountains, and Taiwan Island likely serving as glacial refugia. In the Middle Holocene, suitable areas partially recovered but remained dominated by low to moderate suitability zones. Under current climatic conditions, *Z. schneideriana* achieves its widest distribution, with highly suitable areas concentrated in central and eastern China. Future climate projections suggest a marked reduction in highly suitable areas under high-emission scenarios (SSP585). Centroid shift analysis revealed a persistent northeastward migration from historical periods to the future, reflecting significant ecological adaptability. In light of the habitat contraction risks posed by future climate change, conservation efforts should prioritize the protection of high-suitability areas in central China and the establishment of ecological corridors to facilitate habitat transitions. This study provides a scientific basis for the conservation and management of *Z. schneideriana* and other Tertiary relict species, offering important implications for biodiversity protection.

## Figures and Tables

**Table 1:** Environmental variables used for modeling and contribution values.

Code	Description	Percent Contribution (%)
Bio06	Min temperature of coldest month	21.57
Bio02	Mean diurnal range	19.81
Bio17	Precipitation of driest quarter	13.52
Bio15	Precipitation seasonality (coefficient of variation)	8.32
Bio07	Temperature annual range (Bio05–Bio06)	8.15
Bio12	Annual precipitation	6.58
elevation	Elevation	6.57
Bio18	Precipitation of warmest quarter	6.47
Bio03	Isothermality (Bio02/Bio07) (×100)	5.79
slope	Slope	2.03
Bio08	Mean temperature of wettest quarter	1.04
aspect	Aspect	0.16

**Table 2:** Distribution area changes of *Z. schneideriana* (×10^4^ km^2^).

Period	Scenario	Lowly Suitable	Moderately Suitable	Highly Suitable	Total	Expansion	Unchanged	Contraction
LIG		280.84	13.60	0.00	294.44	107.17	205.35	12.52
LGM		274.54	30.91	0.00	305.45	120.10	207.52	10.35
MH		374.92	43.56	0.00	418.48	219.87	217.68	0.02
Current		41.19	23.85	136.82	201.86			
2050s	SSP126	74.00	43.34	84.58	201.92	16.87	199.58	18.29
	SSP245	81.69	46.45	87.00	215.14	29.59	200.43	17.44
	SSP585	72.03	46.79	92.07	210.88	28.24	197.02	20.86
2070s	SSP126	80.48	42.54	85.44	208.46	20.88	202.69	15.19
	SSP245	96.09	42.78	77.34	216.21	33.89	196.95	20.92
	SSP585	102.06	43.44	65.15	210.66	38.91	184.80	33.07
2090s	SSP126	80.20	39.63	86.41	206.24	24.70	196.28	21.59
	SSP245	109.26	46.88	58.94	215.09	32.70	196.81	21.06
	SSP585	122.74	48.42	41.37	212.53	42.75	182.56	35.31

## Data Availability

The authors confirm that the data supporting the findings of this study are available within the article, and further requests can be directed to the corresponding author.

## Abbreviations

The following abbreviations are used in this manuscript:

IUCN	International Union for Conservation of Nature
VU	Vulnerable
SDMs	Species distribution models
GBIF	Global Biodiversity Information Facility
CVH	Chinese Virtual Herbarium
NSII	National Specimen Information Infrastructure
PPBC	Plant Photo Bank of China
LIG	Last Interglacial
LGM	Last Glacial Maximum
MH	Middle Holocene
CMIP6	Coupled Model Intercomparison Project Phase 6
SSPs	Shared Socioeconomic Pathways
DEM	Digital Elevation Model
NFGIS	National Fundamental Geographic Information System
ANN	Artificial Neural Networks
CTA	Classification Tree Analysis
GBM	Generalized Boosting Model
GLM	Generalized Linear Model
MARS	Multivariate Adaptive Regression Splines
MAXENT	Maximum Entropy
MAXNET	Maximum Network
RF	Random Forest
SRE	Surface Range Envelope
XGBOOST	eXtreme Gradient Boosting
ROC	Receiver Operating Characteristic
AUC	Area Under the ROC Curve
TSS	True Skill Statistic

## References

[1] Fu L., Xin Y., Alan W. (2003). Ulmaceae. Flora of China.

[2] Song Y., Bétrisey S., Kozlowski G. IUCN *Zelkova schneideriana*. The IUCN Red List of Threatened Species 2018: E.T131155456A131155458 2018. https://www.iucnredlist.org/species/131155456/131155458.

[3] Sun J., Qiu H., Guo J., Xu X., Wu D., Zhong L., Jiang B., Jiao J., Yuan W., Huang Y. (2020). Modeling the Potential Distribution of *Zelkova schneideriana* under Different Human Activity Intensities and Climate Change Patterns in China. Glob. Ecol. Conserv..

[4] Zhou Y., Lu X., Zhang G. (2023). Potentially Differential Impacts on Niche Overlap between Chinese Endangered *Zelkova schneideriana* and Its Associated Tree Species under Climate Change. Front. Ecol. Evol..

[5] He J., Jin X., Wu X., Zhang W., Huang C., Zhang Z., Chen Y., Yu Q., Yan W., Wang J. (2024). Environmental Drivers and Conservation Implications of Endangered Ancient *Zelkova schneideriana* Trees in Hunan, China. Biodivers. Conserv..

[6] Araujo M., New M. (2007). Ensemble Forecasting of Species Distributions. Trends Ecol. Evol..

[7] Zhou J., Zhang X., Huo T., Xu H., Meng F., Xu N., Peng C. (2025). Predicting High-Quality Ecologically Suitable Areas of *Astragalus mongholicus* Bunge Based on Secondary Metabolites Content Using Biomod2 Model. Sci. Rep..

[8] Wang R., Guo X., Song Y., Cai Y., Wu Y., Wang M. (2025). Effects of Ultraviolet Radiation as a Climate Variable on the Geographic Distribution of *Oryza sativa* under Climate Change Based on Biomod2. Front. Plant Sci..

[9] Xu Y., Guan B., Chen R., Yi R., Jiang X., Xie K. (2025). Investigating the Distribution Dynamics of the *Camellia* Subgenus *Camellia* in China and Providing Insights into *Camellia* Resources Management under Future Climate Change. Plants.

[10] Zhao Y., Liu J., Wang Q., Huang R., Nie W., Yang S., Cheng X., Li M. (2025). Occurrence Data Sources Matter for Species Distribution Modeling: A Case Study of *Quercus variabilis* Based on Biomod2. Ecol. Evol..

[11] Zhu J., Ji C., Zhang H., Ran Q., Tao S., Wang Z., Xu X., Cai Q., Fang J. (2025). Possible Refugia for Fagaceae Species in China under Climate Change. J. Plant Ecol..

[12] Hu C., Wu H., Zhang G. (2025). Evaluating Habitat Suitability for the Endangered *Sinojackia xylocarpa* (Styracaceae) in China under Climate Change Based on Ensemble Modeling and Gap Analysis. Biology.

[13] Koç D.E., Ustaoğlu B., Biltekin D. (2024). Effect of Climate Change on the Habitat Suitability of the Relict Species *Zelkova carpinifolia* Spach Using Ensembled Species Distribution Modelling. Sci. Rep..

[14] Hijmans R., Phillips S., Leathwick J., Elith J. Dismo: Species Distribution Modeling 2023. https://rspatial.org/raster/sdm/.

[15] Sheppard C.S., Burns B.R., Stanley M.C. (2014). Predicting Plant Invasions under Climate Change: Are Species Distribution Models Validated by Field Trials?. Glob. Change Biol..

[16] Eyring V., Bony S., Meehl G.A., Senior C.A., Stevens B., Stouffer R.J., Taylor K.E. (2016). Overview of the Coupled Model Intercomparison Project Phase 6 (CMIP6) Experimental Design and Organization. Geosci. Model Dev..

[17] Intergovernmental Panel on Climate Change (2021). Climate Change 2021: The Physical Science Basis.

[18] Hijmans R. Terra: Spatial Data Analysis 2020. https://rspatial.github.io/terra/.

[19] Wei T., Simko V. Corrplot: Visualization of a Correlation Matrix 2010. https://github.com/taiyun/corrplot.

[20] Thuiller W., Georges D., Gueguen M., Engler R., Breiner F., Lafourcade B., Patin R., Blancheteau H. (2012). Biomod2: Ensemble Platform for Species Distribution Modeling. https://biomodhub.github.io/biomod2/.

[21] Fielding A.H., Bell J.F. (1997). A Review of Methods for the Assessment of Prediction Errors in Conservation Presence/Absence Models. Environ. Conserv..

[22] Allouche O., Tsoar A., Kadmon R. (2006). Assessing the Accuracy of Species Distribution Models: Prevalence, Kappa and the True Skill Statistic (TSS). J. Appl. Ecol..

[23] Swets J.A. (1988). Measuring the Accuracy of Diagnostic Systems. Science.

[24] Liu Y., Li Y., Wang R., Guo L., Ji Y., Chen Y., Hao L., Lin K. (2025). Impacts of Human Activity and Climate Change on the Suitable Habitats for *Xanthium spinosum* in China. Plants.

[25] Brown J.L., Bennett J.R., French C.M. (2017). SDMtoolbox 2.0: The next Generation Python-Based GIS Toolkit for Landscape Genetic, Biogeographic and Species Distribution Model Analyses. PeerJ.

[26] Yang S., Wang H., Tong J., Bai Y., Alatalo J.M., Liu G., Fang Z., Zhang F. (2022). Impacts of Environment and Human Activity on Grid-Scale Land Cropping Suitability and Optimization of Planting Structure, Measured Based on the MaxEnt Model. Sci. Total Environ..

[27] Yao Y.-F., Song X.-Y., Wortley A.H., Wang Y.-F., Blackmore S., Li C.-S. (2017). Pollen-Based Reconstruction of Vegetational and Climatic Change over the Past ~30 Ka at Shudu Lake in the Hengduan Mountains of Yunnan, Southwestern China. PLoS ONE.

[28] Wang L., Jackson D.A. (2023). Effects of Sample Size, Data Quality, and Species Response in Environmental Space on Modeling Species Distributions. Landsc. Ecol..

[29] Williams J.W., Jackson S.T., Kutzbach J.E. (2007). Projected Distributions of Novel and Disappearing Climates by 2100 AD. Proc. Natl. Acad. Sci. USA.

[30] Zhang Y., Zhang S., Xiao H., Li H., Liao D., Xue Y., Huang X., Su Q., Xiao Y. (2025). Changes in the Distribution Range of the Genus *Cardiocrinum* in China under Climate Change and Human Activities. Biology.

[31] Lin N., Wang Y., Landis J.B., Wang X., He Y., Wang H., Huang X., Liu Q., Yang J., Shang F. (2025). Genome Sequencing and Population Genomics Provide Insights into the Demographic History, Genetic Load, and Local Adaptation of an Endangered Tertiary Relict. Plant J..

[32] Yihui D., Chan J.C.L. (2005). The East Asian Summer Monsoon: An Overview. Meteorol. Atmos. Phys..

[33] Walther G., Post E., Convey P., Menzel A., Parmesan C., Beebee T.J.C., Fromentin J.-M., Hoegh-Guldberg O., Bairlein F. (2002). Ecological Responses to Recent Climate Change. Nature.

[34] Hewitt G. (2000). The Genetic Legacy of the Quaternary Ice Ages. Nature.

[35] Ehlers J., Gibbard P.L. (2007). The Extent and Chronology of Cenozoic Global Glaciation. Quat. Int..

[36] Tian S., Lei S., Hu W., Deng L., Li B., Meng Q.-L., Soltis D.E., Soltis P.S., Fan D.-M., Zhang Z.-Y. (2015). Repeated Range Expansions and Inter-/Postglacial Recolonization Routes of *Sargentodoxa cuneata* (Oliv.) Rehd. et Wils. (Lardizabalaceae) in Subtropical China Revealed by Chloroplast Phylogeography. Mol. Phylogenet. Evol..

[37] Liu X., Huang H., Meng X., Li M., Qin Z. (2025). The Past, Present, and Future Distribution of *Sargentodoxa*: Perspectives from Fossil Record and Species Distribution Models. Ecol. Evol..

[38] Cheng Y., Hwang S., Lin T. (2005). Potential Refugia in Taiwan Revealed by the Phylogeographical Study of *Castanopsis carlesii* Hayata (Fagaceae). Mol. Ecol..

[39] Huang S.S.F., Hwang S., Lin T. (2002). Spatial Pattern of Chloroplast DNA Variation of *Cyclobalanopsis glauca* in Taiwan and East Asia. Mol. Ecol..

[40] Tang C.Q., Matsui T., Ohashi H., Dong Y.-F., Momohara A., Herrando-Moraira S., Qian S., Yang Y., Ohsawa M., Luu H.T. (2018). Identifying Long-Term Stable Refugia for Relict Plant Species in East Asia. Nat. Commun..

[41] Myers N., Mittermeier R.A., Mittermeier C.G., Da Fonseca G.A.B., Kent J. (2000). Biodiversity Hotspots for Conservation Priorities. Nature.

[42] Milne R.I., Abbott R.J. (2002). The Origin and Evolution of Tertiary Relict Floras. Advances in Botanical Research.

[43] Sun H., Zhang J., Deng T., Boufford D.E. (2017). Origins and Evolution of Plant Diversity in the Hengduan Mountains, China. Plant Divers..

[44] Ye X., Ma P., Yang G., Guo C., Zhang Y., Chen Y., Guo Z., Li D. (2019). Rapid Diversification of Alpine Bamboos Associated with the Uplift of the Hengduan Mountains. J. Biogeogr..

[45] Yu S., Zhang J., Li Z., Li W., Ma X., Sun W. (2024). Phylogeography of *Pleurospermum foetens* (Apiaceae) from the Sky Islands of Southwest China. Ecol. Evol..

[46] Zhai T., Wang J., Zhan G., Hu J., Yu L. (2025). Population Genomic Landscapes and Insights for Conservation of the Critically Endangered Island-Endemic Chinese Pangolin in Taiwan. Sci. China Life Sci..

[47] López-Pujol J., Zhang F., Sun H., Ying T., Ge S. (2011). Centres of Plant Endemism in China: Places for Survival or for Speciation?. J. Biogeogr..

[48] Meng H., Su T., Gao X., Li J., Jiang X., Sun H., Zhou Z. (2017). Warm–Cold Colonization: Response of Oaks to Uplift of the Himalaya–Hengduan Mountains. Mol. Ecol..

[49] Chen J., Huang Y., Brachi B., Yun Q., Zhang W., Lu W., Li H., Li W., Sun X., Wang G. (2019). Genome-Wide Analysis of Cushion Willow Provides Insights into Alpine Plant Divergence in a Biodiversity Hotspot. Nat. Commun..

[50] Liu Y., Wang H., Yang J., Dao Z., Sun W. (2023). Conservation Genetics and Modeling Potential Geographic Distribution of *Corybas taliensis*, a Small ‘Sky Island’ Orchid Species in China. BMC Plant Biol..

[51] Cao Y., Comes H.P., Sakaguchi S., Chen L., Qiu Y. (2016). Evolution of East Asia’s Arcto-Tertiary Relict *Euptelea* (Eupteleaceae) Shaped by Late Neogene Vicariance and Quaternary Climate Change. BMC Evol. Biol..

[52] Hewitt G. (1996). Some Genetic Consequences of Ice Ages, and Their Role in Divergence and Speciation. Biol. J. Linn. Soc..

[53] Qiu Y., Fu C., Comes H.P. (2011). Plant Molecular Phylogeography in China and Adjacent Regions: Tracing the Genetic Imprints of Quaternary Climate and Environmental Change in the World’s Most Diverse Temperate Flora. Mol. Phylogenet. Evol..

[54] Song Y., Xu G.-B., Long K.-X., Wang C.-C., Chen R., Li H., Jiang X.-L., Deng M. (2024). Ensemble Species Distribution Modeling and Multilocus Phylogeography Provide Insight into the Spatial Genetic Patterns and Distribution Dynamics of a Keystone Forest Species, Quercus Glauca. BMC Plant Biol..

[55] Jin S., Chi Y., Li X., Shu P., Zhu M., Yuan Z., Liu Y., Chen W., Han Y. (2023). Predicting the Response of Three Common Subtropical Tree Species in China to Climate Change. Front. For. Glob. Change.

[56] Pearson R. (2006). Climate Change and the Migration Capacity of Species. Trends Ecol. Evol..

[57] McLeman R., Smit B. (2006). Migration as an Adaptation to Climate Change. Clim. Change.

[58] Thomas C.D. (2010). Climate, Climate Change and Range Boundaries. Divers. Distrib..

[59] Feng C., Zhang J., Huang H. (2023). *Parallel situ* Conservation: A New Plant Conservation Strategy to Integrate In Situ and Ex Situ Conservation of Plants. Biodivers. Sci..

[60] Morelli T.L., Maher S.P., Lim M.C.W., Kastely C., Eastman L.M., Flint L.E., Flint A.L., Beissinger S.R., Moritz C. (2017). Climate Change Refugia and Habitat Connectivity Promote Species Persistence. Clim. Change Responses.

[61] Volis S. (2019). Conservation-Oriented Restoration—A Two for One Method to Restore Both Threatened Species and Their Habitats. Plant Divers..

[62] Zeren Cetin I., Ozel H.B., Varol T., Canturk U., Sevik H. (2025). Climate Change Impacts on *Taxus baccata* Distribution and Conservation. J. For. Res..

[63] Yan F., Wei T., Yang C., Yang Y., Luo Z., Jiang Y. (2024). Combined Analysis of Untargeted Metabolomics and Transcriptomics Revealed Seed Germination and Seedling Establishment in *Zelkova schneideriana*. Genes.

